# The Present and Future of Pain Management in Patients With Chronic Kidney Disease: A Narrative Review

**DOI:** 10.7759/cureus.100490

**Published:** 2025-12-31

**Authors:** Igor Wilderman, Francesca Sarzetto, Budvin Wijetillake, Sydney Verdun

**Affiliations:** 1 Pain Management, Wilderman Medical Clinic, Thornhill, CAN; 2 Pain Management, Canadian Centre for Clinical Trials, Thornhill, CAN; 3 Research, Canadian Centre for Clinical Trials, Thornhill, CAN

**Keywords:** analgesia, anesthesia, chronic kidney disease, chronic pain, pain management

## Abstract

Chronic kidney disease (CKD) is an increasingly common ailment, greatly affecting the quality of life, morbidity, and mortality of the population. In addition, more than half of patients with CKD experience chronic pain, mostly of nociceptive or neuropathic origin, and physicians frequently have to find a difficult balance between safety and efficacy to control it. The altered metabolism and renal excretion in patients with CKD modify the pharmacokinetics and pharmacodynamics of several analgesic drugs, making this population more prone to side effects and lower efficacy, since the doses often need to be reduced. This narrative review describes the current pharmacological approaches for nociceptive and neuropathic pain, and emerging alternatives, such as cannabinoids and low-dose naltrexone. We also describe the current knowledge on anesthesia, perioperative and acute pain management, and injectables, including ketamine and corticosteroids (intra-articular and epidural). In the variable and possibly deteriorating clinical context of CKD, this review shows that pain management needs to be individualized and carefully discussed with the patient; close monitoring is also necessary to adjust the treatment and obtain effective pain control while minimizing the risk.

## Introduction and background

Chronic kidney disease (CKD) is a major global health concern, affecting an estimated 13% of the worldwide population, approximately 6% in stages 3-5 (more than 800 million individuals, varying with the definition and specific diagnostic criteria considered), and it is one of the leading causes of mortality worldwide [1]. The prevalence of CKD has increased significantly in the last two decades, due to the aging population and the increasing burden of chronic conditions like hypertension, diabetes, and obesity [1]. In advanced stages of CKD, the survival outcomes can be improved by some treatment options, such as dialysis (peritoneal or hemodialysis). Older patients with Stage 5 CKD (glomerular filtration rate (GFR) < 15 ml/min/1.73 m^2^) who opted for dialysis have shown an 84% survival rate at year one and 76% at year two, whereas those treated conservatively experienced lower survival rates at corresponding times after the intended dialysis start date of 68% and 47%, respectively. However, in patients with several comorbidities, the survival rate is similar to that of patients not undergoing dialysis [2]. Such comorbidities may cause or be caused by CKD, or be independent, and with the addition of mental health disorders they can increase the risk of hospitalization and mortality; a study reported that 25% of patients with CKD have three or more comorbidities, and the most common and dangerous are cardiovascular conditions (e.g., hypertension, stroke, atrial fibrillation, heart failure) and diabetes [3].

The majority of patients with CKD and its most severe form, end-stage renal disease, experience chronic pain; this symptom has been reported in more than 50% of all patients and 60-90% of those undergoing dialysis, and 75% of the latter report inadequate pain management [4,5]. This condition can result in reduced quality of life (QoL), reduced compliance with dialysis treatment, increased healthcare service utilization, and higher mortality rates [6]. Chronic pain is also often associated with mental health alterations, including depression and anxiety [6], sleep disturbances, and reduced life satisfaction [7]. Moreover, alterations of physical and cognitive functional status, apathy, and reduced QoL are associated with increased mortality among elderly patients with CKD, particularly those in advanced stages of the disease [8].

The pain associated with CKD is multifaceted; often it is nociceptive, due to musculoskeletal or systemic disorders, such as osteoporosis or amyloidosis due to hemodialysis (HD), or independent, while ischemic (peripheral vascular disease) and neuropathic pain (polyneuropathy) are also frequent; each type can present isolated or in combination [4,9]. In addition, patients undergoing HD often experience pain due to the procedure itself, such as access-related soreness, muscle cramps, and headaches [10], and effective pain management requires an accurate diagnosis to identify its main source(s) and choose medications accordingly [11]. A wide variety of possible treatments is available, and each has variable effects in different patients, or in the same patient at different times; therefore, pain management often requires a trial-and-error approach and monitoring [12]. Furthermore, higher intensity and frequency of uncontrolled pain can lead to increased use and possibly abuse of analgesics, causing iatrogenic alterations in addition to the pathologic burden of these patients [13].

The purpose of this narrative review was to summarize the current options for the management of chronic pain in patients with CKD, including their risks and benefits in this population, and describe possible alternatives that emerged in recent years with promising results, though still needing further research. In addition, we focused on the specific challenges of chronic pain management in CKD, and also described the pharmaceutical options for anesthesia and acute pain management, with the aim of providing a comprehensive guide for various physicians treating these patients.

## Review

Current pain management options for patients with CKD

Pain management in patients with CKD requires careful considerations, due in primis to the reduced urinary excretion of drugs, and also to other possible pharmacokinetic alterations in absorption, volume of distribution, and metabolic elimination; therefore, analgesic doses typically need to be lower than the standard ones [5]. On the other hand, some drugs are eliminated quickly during HD and may require supplemental doses after each session [5]. In addition, pain management should set realistic goals and be discussed with the patient; it is often impossible to eliminate the pain completely, whereas a 30% intensity reduction is generally considered an attainable target that can sensibly improve their functionality and QoL [11]. For this review, we searched PubMed for articles describing the general pain management strategies for patients with various stages of CKD, for chronic and acute pain, nociceptive and neuropathic, and more in detail for specific classes of drugs, or specific drugs, and their risks, benefits, and challenges in this population, including patients undergoing HD. We used various sources to compare the general concepts and also expanded the search through the bibliography of the articles identified. 

The typical treatment approach for CKD-associated pain follows a stepwise strategy, according to guidelines from the World Health Organization adapted for these patients, effective at least in the short term for acute pain [6,9,14,15]. In addition, for successful management, it is critical to determine whether the pain is mostly nociceptive or neuropathic, the two main categories, though each treatment can have vastly different effects in different patients.

Non-pharmacological approaches may be attempted first in all cases, including cognitive-behavioral therapy, relaxation, music therapy, exercise, yoga, aqua therapy, or physical/thermal therapy; these options are generally considered safe, though the evidence of their efficacy on CKD pain is limited [6,15]. These measures can also be used in conjunction with other adjuvant treatments (i.e., drugs with primary indications different from pain), like antidepressants or antiepileptics, for neuropathic pain (possibly effective on their own or as support to analgesics), and all these options can be used in addition to the main analgesia at every step. Step 1 is indicated for mild or acute pain (intensity rated as 1-3 on an 11-point numerical rating scale (NRS), which goes from 0, no pain, to 10, the worst pain imaginable). It typically involves non-steroidal anti-inflammatory drugs (NSAIDs) for nociceptive pain, although most of them are nephrotoxic and should be used only for short periods (≤ 5 days) or in patients with mild CKD, and anticonvulsants for neuropathic pain. In Step 2, for moderate or persisting pain (NRS 4-6), a low dose of opioid can be added, and for severe pain (NRS 7-10), opioids may be considered in larger doses (Step 3) [14].

NSAIDs and opioids are the most commonly used analgesics to manage pain associated with CKD. A systematic review estimated that approximately 40% of patients with CKD regularly use analgesia, with 50% of these taking opioids and 21% NSAIDs (and respective chronic use at 20% and 7%), whereas fewer data are available on the use of antiepileptics [16]. However, more recent guidelines from the USA and Canada suggest a cautious use of opioids, only when non-pharmacological and non-opioid therapies failed to control the pain, and only when the expected benefits for a patient outweigh the risks [17,18].

Non-Steroidal Anti-Inflammatory Drugs and Acetaminophen

For various types of mild nociceptive pain, NSAIDs are usually considered first [15]; however, NSAIDs in general and even selective COX-2 inhibitors can be harmful in patients with CKD, due to their known nephrotoxicity, possibly increased risk of gastrointestinal bleedings, and other complications, including acute kidney injury, hypervolemia, hyperkalemia, hyponatremia, and hypertension [19]. A review reported that in general, NSAIDs are correlated with a higher risk of hospitalization, whereas the association between NSAID use and risk of gastrointestinal bleedings or CKD progression was found to be not significant, in contrast to previous reports [20-22]. They also reported that non-selective NSAIDs are associated with a greater risk of stroke and other major cardiovascular events [16]. These risks are particularly high with prolonged use or in patients with Stage 3-5 CKD [5,15,23,24], and lower in patients with Stage 1-3 CKD, under 65 years of age, and without other severe comorbidities, such as congestive heart failure, nephrotic syndrome, and cirrhosis [19]. Use of NSAIDs can be considered generally acceptable for short periods (≤ 5 days) or longer in carefully selected patients, with close monitoring of renal function. In Stage 4, they could still be used for short periods with the same precautions, except in patients with an underlying risk of hyperkalemia, whereas for patients in Stage 5, they should be used only for palliative care [19]. Studies have shown a similar risk of acute kidney injury across all NSAID agents, though oxicams, like meloxicam and piroxicam, oxaprozin, and ketorolac, are associated with higher risks, whereas ibuprofen and indomethacin have lower risks even at standard dosages [19,25]. COX-2 inhibitors are associated with a 33% increased cardiovascular risk, whereas ibuprofen poses some cardiovascular risk at high doses and a lower risk of gastrointestinal bleeding at lower doses [26]. In contrast, acetaminophen and aspirin are safe individually even for patients in Stage 4 and 5 [27], though the concomitant use of these two drugs may accelerate CKD progression [21].

If prolonged treatment is needed, acetaminophen and topical NSAIDs should be suggested as a safer alternative [6], considering also that several patients self-medicate with NSAIDs, unaware of their side effects, since these drugs are available over the counter [23]. However, topical NSAIDs were still found to be significantly associated with adverse renal outcomes in patients with CKD, though with reduced frequency and severity compared to oral NSAIDs, and the cardiovascular risk is reportedly 36% lower with topical than with oral NSAIDs [28]. On the other hand, inadequate pain management is also a negative prognostic factor for this population, increasing the incidence of depression and anxiety [6]; the risks of NSAID overdosing should be assessed for each patient and compared to the possible risks and benefits of other drugs, such as opioids or gabapentinoids for neuropathic pain [29].

Antiepileptics

Antiepileptics are effective particularly for the neuropathic pain caused by diabetes or post-herpetic neuralgia, whereas the evidence is limited for CKD [12]. The antiepileptic gabapentinoids (pregabalin and gabapentin) are often prescribed to increase the inhibitory activity of gamma-aminobutyric acid (GABA) and thus suppress neuropathic pain, and they are generally well tolerated in patients with CKD [5,6]. However, the dosage needs to be lowered for these patients, as the drug excretion is mainly urinary, and conversely, supplemental doses may be needed after HD sessions, due to their rapid elimination during this process [5]. On the other hand, a previous study reported that initiating gabapentinoid therapy at normal doses in patients with CKD had only a minimal increase in adverse events (encephalopathy, falls, fractures, or respiratory depression) in the first 30 days compared to initial lower doses [30]. In patients with decreasing GFR, and particularly those undergoing HD, the doses need to be carefully monitored and adjusted, as toxicity has been reported for serum levels of gabapentin > 15 µg/mL, and a small group of HD patients was found with concentrations almost four-fold this value [31]. Furthermore, other studies with large samples showed consistent associations between high-dose gabapentinoid use and several mental status alterations in patients with CKD [16,30,32].

Symptoms of gabapentin toxicity include tremulousness, confusion, dizziness, myoclonus, ataxia, and reduced consciousness; elderly patients with renal dysfunction and several co-morbidities are more at risk of toxicity and should be monitored closely [31]. In patients undergoing HD, both gabapentin and pregabalin were found to be associated with a higher risk of altered mental status and falls, even at low doses [32]. In contrast, carbamazepine, another anticonvulsant with a chemical structure similar to tricyclic antidepressants (TCAs), may be as effective as gabapentin for neuropathic pain with fewer side effects, and it does not require dose adjustments in patients with CKD [12].

Muscle Relaxants

Other GABA agonists are the muscle relaxants baclofen and tizanidine, used to treat spastic movement due to motor-neuron disorders, whereas antispasmodics like cyclobenzaprine (similar to TCAs in structure) treat musculoskeletal spasms [33,34]. Both drug types may be used for muscular pain, cramps, or hiccups (depending on their origin), particularly in patients undergoing HD [33,34]; however, baclofen in particular is associated with an increased risk of neurotoxicity (e.g., encephalopathy, respiratory depression, unconsciousness), even in subjects with preserved renal function, a risk significantly higher than those associated with cyclobenzaprine or tizanidine use [35]. Baclofen is mainly excreted by the kidneys; thus, in patients with CKD, it has a prolonged half-life, and in particular in patients with hypoalbuminemia [33], its free form is more abundant and can penetrate the blood-brain barrier, causing neurotoxicity even at subtherapeutic dosages [36]. A study reported a neurotoxicity incidence of 7% among patients with CKD Stage 4-5 or undergoing HD and suggested reduced dosages for these patients [33]. Tizanidine has similar efficacy and is considered safer for these patients, still with reduced dosages, as it is associated with less severe side effects, though it can cause drowsiness more often; additionally, it reduces neuropathic pain and has a gastroprotective effect that can increase the tolerance of NSAIDs [37]. However, a large study among HD patients using various muscle relaxants (10% of all HD patients, with cyclobenzaprine the most commonly prescribed, in approximately two-thirds of patients) reported an increased risk of falls and fractures due to muscle weakness [34]. Therefore, muscle relaxants should be used with caution, with a preference for tizanidine for motor-neuron disorders and cyclobenzaprine for muscular spasms, and monitoring of the muscular function.

Antidepressants

Another pharmacological option for neuropathic pain is antidepressants, particularly serotonin-norepinephrine reuptake inhibitors (SNRIs), as norepinephrine acts as the main analgesic mediator, and TCAs, like amitriptyline [5,12]. TCAs, like gabapentinoids, are effective in particular for diabetic and postherpetic neuropathic pain; however, no specific extensive evidence was found in the literature on the efficacy of antidepressants for pain relief in patients with CKD. Moreover, they may cause more side effects than the anticonvulsants, like dry mouth, orthostatic hypotension, and drowsiness [12]. Amitriptyline is metabolized by the liver and not dialyzed; therefore, it can be administered at normal dosages in patients with CKD, though lower dosages are typically sufficient and reduce the risk of side effects in this population [12]. SNRIs are excreted renally, and therefore the dose should be reduced in patients with CKD; nonetheless, they are generally well tolerated and considered safer than the older TCAs, thanks to their closely targeted action and lower risk of side effects [5]. However, an exception is duloxetine, as its plasma levels were reportedly increased in patients with end-stage renal disease in a pharmacokinetic study, and therefore the drug is contraindicated in these patients [38]. In addition, these drugs seemingly have limited or no efficacy in reducing depressive symptoms in patients with CKD, due to the accumulation of uremic toxins causing treatment resistance [39]. Overall, antidepressants may help reduce neuropathic pain in the CKD population, although the evidence is insufficient to consider them as main analgesics. 

Opioids

Opioids are strong analgesics, commonly used for moderate-to-severe chronic pain; they are particularly effective for nociceptive pain, but they can also be used for neuropathic alterations (e.g., diabetic neuropathy) [6]. Some physicians even consider them the first line of treatment in patients with CKD, due to their general effectiveness and lower risk of nephrotoxicity and gastrointestinal bleeding compared to NSAIDs [5,40], though they can cause severe side effects, and caution is usually required. Opioids are typically started for short-term treatment, regardless of renal status, though 3-5% of patients continue taking these drugs in the long term (> 3 months) [40]. Studies on the effectiveness of long-term treatment with opioids are lacking, particularly for the CKD population [18, 40]; however, a review reports at least partially effective pain relief in 60-80% of patients with end-stage renal disease [41]. On the other hand, 8-12% of patients undergoing chronic use develop opioid use disorder, defined as use leading to serious impairment or distress [40].

Furthermore, another study reported higher risks of kidney failure and hospitalization with chronic use of opioids compared to NSAIDs, particularly in individuals of black ethnicity, the elderly, or with diabetes [42]. In addition, opioids can have significant dose-dependent side effects; common alterations are drowsiness, dizziness, vomiting, urinary retention, and, most frequently, constipation. Moreover, they can induce altered mental status, risk of dependency and overdose, respiratory depression, and cardiac rhythm alterations, with significant increases in morbidity and mortality among their users [40]. In particular, respiratory depression is a life-threatening event, though uncommon in patients without co-morbidities using opioids only for chronic pain management; if necessary, it can be reversed with naloxone [43]. The reduced kidney function in CKD can cause drug accumulation, since after hepatic metabolization opioids are eliminated in the urine, increasing the risk of toxicity and potentially worsening kidney damage in this population [15,40]. Therefore, they should be used with caution, only when other options are ineffective, and possibly in combination with other options, pharmaceutical or not [6]. They should be approached carefully by both patients and physicians, guided by informed and shared decision-making [40].

If patients with CKD experience side effects, the clinicians should consider reducing the dose or frequency of administration, choosing a different opioid, or selecting an alternative analgesic. In practice, however, opioids are often overprescribed, particularly in patients with end-stage renal disease or undergoing HD, with a high risk of side effects even at low doses [40,41,43,44]. Previous studies reported variable prevalence of use, with a review of 15 studies from 12 countries citing use in5 5-36% of patients with end-stage renal disease [41], whereas a study from the USA reported opioid use in 64% of HD patients [44]. In fact, they can be useful tools for selected patients, improving the QoL and general functionality [6,40], though 17-50% of patients using opioids still experience moderate or severe pain, particularly those suffering from neuropathic pain [40]. On the other hand, for patients with end-stage CKD in the context of palliative care, pain should be controlled as effectively as possible, and opioids should not be withheld [40]. 

Opioid use needs to be carefully monitored, with initially weekly and then monthly follow-ups [18]. A personal or family history of substance addiction increases the risk of misuse and abuse, and some mental illnesses (e.g., depression, bipolar disorder, attention-deficit disorder, schizophrenia) may also increase the risk of opioid abuse [11,40]. In all of these conditions, the Canadian guidelines recommend against proposing a trial of opioids to the patient [17], whereas the American guidelines suggest a careful discussion with at-risk patients before starting opioid therapy. The discussion should include thoroughly explaining risks of opioid abuse and overdose, offering naloxone to reverse the effect of a possible overdose, frequent monitoring, frequently suggesting alternative therapies or opioid tapering in case of long-term use, and multidisciplinary support, with physical therapy and psychological help [18]. Moreover, these guidelines suggest an initial discussion with the patient about the expected risks and benefits of opioids on pain and function, establishing treatment goals and a safe exit strategy in case the drugs are not effective or not tolerated, and particularly close monitoring of patients with renal or hepatic impairment [18].

The therapy should be initiated at low doses, avoiding extended-release or long-acting formulations in opioid-naïve patients, proceeding with slow dose increases to establish the minimum effective dose, and carefully monitoring for side effects, particularly when increasing the dose or switching to a different drug [11,18,40]. The choice of a specific opioid, in fact, is also important: tramadol (a synthetic opioid also working as an SNRI) and oxycodone can be used for moderate pain, and hydromorphone, fentanyl, methadone, and buprenorphine are considered safer than others for severe pain in patients with CKD [9]. Oxycodone is one of the most commonly prescribed opioids for this population, whereas morphine and codeine should be avoided, due to accumulation of toxic metabolites, neurotoxic effects, and respiratory depression [6,40,43], and also increased risk of falls and fractures [44]. Methadone and buprenorphine are typically used for the treatment of opioid use disorder; they can also be useful for CKD-related chronic pain, with low risk of overdose. However, methadone can prolong the cardiac QT interval and cause other ventricular arrhythmias, and should be used only at reduced dosages [6,40]. Buprenorphine is considered safe without dosage adjustments, as it is metabolized by the liver and excreted in the bile [43]. Additionally, it can also be administered through dermal patches to delay the first-pass hepatic metabolization [40,43], and transdermal administration of buprenorphine or fentanyl, starting at half the normal dosage, seems to be the treatment of preference [43].

In patients undergoing HD, all opioid types are correlated with a small risk of hospitalization from overdose, ranging from moderate increase (hydromorphone) to very high increase (methadone, buprenorphine, oxymorphone) compared to non-users, with the exception of hydrocodone (lower risk); overall, the annual risk is < 0.2% in patients with one or more opioid prescription [45]. In addition, concomitant use of opioids and benzodiazepines or gabapentinoids should be avoided, as these combinations greatly increase the risk of opioid overdose, respiratory depression, and hospitalization [40,45]. Some physicians are hesitant to prescribe opioids for chronic pain management, regardless of CKD status, due to concerns about side effects, abuse, and addiction [41]; therefore, alternative pain management strategies for CKD patients are needed.

Corticosteroid Injections

Intra-articular or epidural injections of corticosteroids are common treatments for osteoarthritis or low back pain; however, studies on their safety and efficacy in patients with CKD who also suffer from these ailments are lacking. The only evidence regarding this population was found in a small study comparing the efficacy and infection risk of knee steroid injections in two groups of 20 patients each, undergoing HD or not (23 and 24 knees, respectively), though it was not specified whether the non-dialysis patients had preserved renal function [46]. Both groups showed significant improvements in pain intensity at 3 and 6 months, and the non-HD group had larger improvements in functionality, though the study only compared each group with its baseline values and not with the other group. No infection occurred in either group, which is not surprising, considering that the reported rate of intra-articular infections after cortisone injections is lower than 1/100,000 [47]; thus, assessing this risk in small groups of possibly immunocompromised patients with CKD is challenging.

In the general population, known immediate systemic side effects from musculoskeletal steroid injections include adrenal suppression, headache, transient hypertension, postprandial hyperglycemia, and psychiatric alterations, the latter more common in elderly patients with reduced renal function; possible delayed effects include adrenal insufficiency and osteoporosis [47]. Corticosteroids should be administered with caution in patients with diabetes, whereas CKD is not mentioned as a contraindication [47]. On the other hand, systemic steroid therapies (oral or intramuscular) in patients with primary proteinuric kidney disease were reported to be frequently associated with adverse events, as 62% of the patients treated (approximately 30% per year) presented one or more side effects, including hypertension (17% per year), diabetes (8%), obesity (7%), overweight (6%), and any infection (5%) [48]. However, the side effects after intra-articular injections are less frequent and less severe than after systemic therapies, even when administered in several joints at the same time [49].

In addition, reducing overactivation of the renal sympathetic nerves with epidural anesthesia could play a significant role in slowing CKD progression [50]. A study induced CKD in rats through partial nephrectomy and aimed to determine the impact of epidural injections with lidocaine (administered as a bolus followed by infusion, 6 hours daily for a week) to reduce the sympathetic nerve activity and possibly treat CKD [51]. Lidocaine reduced the renal sympathetic nerve overactivity, contributing to blood pressure normalization (a critical factor, as cardiovascular complications are a major driver of morbidity and mortality in CKD [3]) and decreased sympathetic-driven renal damage. It also improved the renal function, as shown by reduced serum creatinine, proteinuria, and markers of tubular damage. Histological analysis showed reduced fibrosis and structural kidney damage in treated rats [51]. These findings suggest that epidural anesthesia could serve as a novel treatment to stop or delay CKD progression while also alleviating pain. However, the detailed biological mechanism of epidural lidocaine block inducing these therapeutic effects and how these findings would translate to humans remains unknown.

Emerging adjunctive and experimental options

Adjunctive analgesic medications like cannabinoids [52-71] and low-dose naltrexone [72-77] have shown promising results for the management of chronic neuropathic pain and may be considered for patients with CKD. However, previous studies reported wide variability in effectiveness and safety for both types [52,54,62-67,75,76], possibly due to the lack of standardized protocols for dosages, frequency, and routes of administration, and the use of these drugs in this specific population remains to be investigated [52-54,57,62,68,73].

Cannabinoids

Recently, cannabis and its derivatives (natural and synthetic) have been attracting attention as a possible alternative or adjunct therapy to opioids for chronic pain relief, though evidence in patients with CKD is lacking, as they showed some good results in other clinical contexts [52,53]. These drugs interact with receptors of the endocannabinoid system, found mainly in the central nervous system, reducing pain and inflammation, in addition to their known psychoactive effects [54]. Cannabinoids are available in a wide variety of formulations, dosages, and routes of administration, and are under legal concerns, depending on the country of interest; therefore, strong evidence about their efficacy is lacking [52,54,55]. Most of the studies available addressed the general efficacy of cannabinoids on chronic pain of several origins, and these drugs seem to provide a moderate-to-good relief for neuropathic pain [56,57], particularly when administered by inhalation [56,58]. In addition, other studies reported significant pain relief for musculoskeletal pain, as noted in a recent review [54], as well as improved physical functioning and sleep quality [59], increased appetite, and lower anxiety [60].

Limited evidence suggests that cannabinoids may be not-inferior to opioids, providing effective relief for chronic pain in approximately half of the patients [54,61], and they are associated with fewer and less severe side effects than opioids [55]. However, the results reported are highly variable [52,62], and a large overview of 57 systematic reviews found that most studies provided their positive (25 reviews) or negative recommendations (12 reviews) about cannabis-based medicine based mostly on evidence considered of very low or low quality [63].

In addition, side effects are relatively common [52,57,64]. Mostly, they are temporary and mild [52,56], though involving several organs, including the brain and central nervous system (psychiatric and cognitive symptoms: e.g., dizziness, drowsiness, confusion, anxiety), gastrointestinal (nausea, vomiting, constipation), cardiovascular (palpitations), respiratory (cough, dyspnea), sensory (vision, hearing alterations), and other systems. The incidence of these side effects is highly variable in clinical studies, resulting again in low-quality evidence [65]. Severe adverse events are reportedly rare; however, they include psychosis, dependency, increased mortality due to myocardial infarction, tumors (associated with smoked cannabis, as for cigarette smoking), and increased probability of motor vehicle accidents [52,65]. Moreover, a long-term study showed that after 4 years of cannabinoid use, patients had more severe scores for pain and anxiety compared to non-users [66], though long-term evidence is sparse [54,57]. Recently, a comprehensive review for the general treatment of neuropathic pain gave a weak negative recommendation against cannabis products [67], though the clinical trials analyzed specifically for these drugs were only 18.

For patients with CKD, the evidence is even more sparse [52,53], as no studies focused on this population and long-term use [54,57]; however, these products seem not to have negative effects on the renal function [68,69], though a study reported a faster GFR decline in users than in non-users [70]. Cannabinoids are mainly metabolized in the liver and excreted in the feces, and only 20-35% is eliminated in the urine; they have a large volume of distribution and are unlikely to be eliminated by HD [52]. They may be offered to patients with CKD on an individual basis in case of treatment-resistant pain and low individual risks, starting at low doses and titrating, preferably in safer oral forms (tablets or sublingual oils) rather than inhaled formulations [62,68], and only after careful patient selection and discussion [54]. A survey of Canadian nephrologists showed that the majority of these physicians feel they have insufficient knowledge of this option and currently prefer to avoid prescribing cannabinoids; however, they would reconsider their usage if more solid evidence of efficacy and safety in patients with CKD became available [71]. Further research on cannabinoids is warranted.

Low-Dose Naltrexone

A relatively new option to treat chronic neuropathic pain gained increasing interest in the last couple of decades: low-dose naltrexone (LDN). This drug is an opioid antagonist, similar to naloxone, and is typically used to treat opioid and alcohol addiction; however, at approximately 1/10 of the dosage for addiction, it behaves similarly to opioids, with paradoxical analgesic and anti-inflammatory properties, absent at the larger standard doses [72]. Two distinct mechanisms of action are possibly involved to cause the opposite behaviors: a rebound effect after temporary blockage by LDN, increasing the number and sensitivity of opioid receptors, and inhibition of an independent pro-inflammatory receptor, present in microglia and macrophages in the central nervous system [72,73]. Thus, LDN acts as a glial cell/ neuroimmune modulator.

Small clinical studies demonstrated the safety and efficacy of LDN in chronic pain and inflammation diseases, such as fibromyalgia, multiple sclerosis, Crohn’s disease, and diabetic neuropathy (often comorbid with CKD) or other types of neuropathies [72-74]. In addition, a large review reported generally positive results in several dermatologic conditions, cancer pain, post-COVID-19 syndrome, and other autoimmune and inflammatory disorders [75], whereas other studies reported only minimal benefits [75,76]. However, a peculiarity of LDN is the difficulty in determining the optimal dosage, as demonstrated by one study with thorough titration, which showed effective dosages varying between 0.1 and 5.6 mg/day in different patients with a linear distribution [77], and the benefits emerged only after 1-6 months of use [72,74]. Therefore, clinical studies should evaluate several dosages over long periods of time to estimate their effectiveness properly.

On the other hand, an advantage of LDN is its safety, as no severe side effects, chronic toxicity, or withdrawal symptoms were reported; mild side effects include gastrointestinal symptoms, headache, and sleep modifications, including possible improvements and vivid dreams [72,76,77]. Moreover, it is inexpensive and easily accessible, although commercially available currently only at the higher dosages for addiction treatment (50-100 mg); therefore, LDN must be provided by a compounding pharmacy or self-compounded by the patient [72,76]. Larger clinical trials are warranted to determine LDN efficacy in additional conditions, including chronic CKD pain, and identify the most appropriate dosages, as it is a promising alternative to opioids. In patients with CKD, the dosages might need to be further adjusted, as LDN is excreted primarily in the urine after hepatic metabolization, though mild renal impairment reportedly did not require dosage modifications [73]. Furthermore, LDN may be useful in this population as it also decreases pruritus, another common and challenging CKD symptom, though this effect was reported for other disorders [75].

Ketamine Infusions

Ketamine is an anesthetic drug that has analgesic functions at lower dosages, useful in cases of neuropathic pain resistant to other treatments, as well as acute pain [12,78]. It can be used in patients with CKD without dose adjustments due to its short half-life; it is metabolized by the liver and excreted in the urine [78]. The intravenous route (as bolus or infusion) is the most beneficial to maximize the bioavailability and reduce the risk of abuse; moreover, possible side effects cease quickly after infusion termination [78]. However, its use is controversial, due to hallucinogenic and dissociative properties that make it subject to abuse as a recreational drug; it may cause severe side effects at higher doses, like tachycardia and psychosis. These adverse events can be prevented with concurrent administration of haloperidol, clonidine, or midazolam, whereas magnesium can potentiate its analgesic effects [12,78]. Low-dose ketamine can be useful in critically ill patients as a substitute for opioids, reducing hyperalgesia and allodynia in chronic pain; in addition, it has an anti-depressive effect, thanks to its mechanisms of action involving different signaling pathways [78]. Its use as an alternative analgesic has been increasing in the last few years due to the opioid crisis, especially in emergency medicine and acute pain, and physicians treating chronic pain should also consider it as a feasible option, though more research is needed for efficacy and safety [12,78].

Challenges to treating chronic pain in patients with CKD

Chronic pain management requires an individualized and comprehensive approach that starts with careful assessment of the type of pain, its underlying causes, intensity, and duration. An effective treatment of this symptom in patients with CKD can help mitigate the disease burden, reduce hospitalizations, and ultimately improve QoL and survival rates [6]. However, CKD-related pain is multifaceted and rarely due to a single cause, and its varying intensity and nature contribute to heterogeneous clinical pictures. This variability can lead to misdiagnosis, inadequate treatment, and inconsistency of findings between studies, hindering the standardization of effective treatment strategies. In addition, a systematic review noted that most research is based on scales validated to assess general chronic pain, rather than specific tools for neuropathic pain, resulting in potentially underestimation or inaccurate prevalence rates for this type of pain [79].

Furthermore, robust clinical trials and evidence on the safety and efficacy of pharmacological pain treatments are often lacking for patients with CKD, possibly due to flaws in trial design, analysis, and reporting [80]. The results need to be interpreted cautiously, as improvements also occurring in control groups (administered a placebo or a different medication) can skew the perception of intervention effectiveness. Furthermore, the scarcity of evidence can lead to uncertainty regarding the most effective treatment options and guidelines for this population; a multimodal, individualized approach should be followed, based on the type and severity of pain, CKD stage, co-morbidities, and other patient characteristics [15]. 

Several common analgesic medications, such as NSAIDs and some opioids, can increase the risk of CKD progression [81]. Despite these risks, or unaware of them, patients and physicians often use NSAIDs and opioids to manage the pain, considering the limited effective alternative options, and possibly administering them at higher doses than recommended in this population, developing abuse and drug-related problems [13,23,40,44]. Antiepileptics may also be toxic and cause severe side effects [30-32]. The delicate balance between effectiveness and toxicity creates uncertainty about pain management and frustration for the patients, who often report that the pain is not managed effectively [5,11]. The physical and emotional tolls highlight the need for improved pain management strategies tailored to the unique challenges faced by CKD patients; even those with no chronic pain may use NSAIDs and other drugs unsafely [13].

The nature and severity of pain can change over time, necessitating frequent reassessments and adjustments of treatment strategies. The patients may also develop additional comorbid conditions as CKD progresses, which may exacerbate the pain, further complicating its management and accelerating disease progression. It was reported that 25% of patients with CKD have three or more comorbid conditions, and 7% have five or more [3]; overall, these patients could take 10-12 different medications concurrently, between prescriptions and over-the-counter ones, for a total average of 19 pills per day [40]. This complex dynamic can create significant challenges for healthcare providers, as they must navigate the shifting clinical picture of a patient’s symptoms and overall health status to provide long-term effective pain management and maintain an adequate QoL.

Furthermore, mood disorders such as depression, anxiety, and apathy are highly associated with CKD [8,39,82], and chronic use of antidepressants is higher among these patients than in the general population [83]. These issues can also exacerbate the pain and further reduce functionality and general energy, resulting in lower QoL. Addressing psychopathological conditions requires a multidisciplinary approach, combining non-pharmacological options, antidepressants, and pain management medications, while considering the possible drug interactions. Alternative options include behavior-based (e.g., cognitive-behavioral therapy, relaxation, music therapy) and physically oriented interventions (e.g., exercise, physical therapy, thermal therapy, transcutaneous electrical stimulation) [6,15]. However, the evidence of efficacy for these treatments is limited and mostly focused on acute musculoskeletal pain in the general population [15]. Discussing realistic expectations for pain control and functional improvements is also particularly important for patients with anxiety and depression, as mismatched expectations can increase the emotional distress [6,11].

Anesthesia, peri-operative, and acute pain

Local and general anesthetics are commonly used in patients with CKD to manage acute procedural or perioperative pain, for interventions such as dialysis catheter insertions, arteriovenous fistula creation for HD, dental procedures, kidney biopsies or surgery, including transplantation [84]. Anesthesia can be administered through a variety of routes, from intrathecal or peridural to intravenous, inhalatory, and transdermal routes [84]. General anesthesia is often performed in these patients; however, it requires careful pre-operative assessment and possibly interventions, including HD performed 6-24 hours before surgery for optimal electrolyte and volume levels, medications to reduce anxiety, and then close intra-operative monitoring [85]. Alternatively, epidural anesthesia and regional nerve blocks with bupivacaine and lidocaine can be administered to reduce risks in these patients even in kidney transplants [85], with effectiveness and safety comparable to general anesthesia [84], lower risk of hyperkaliemia [86], less bleeding, and improved post-operative pain relief, provided by epidural infusion controlled by the patient [85]. Among intravenous anesthetics, propofol shows a decreased time between infusion cessation and eye opening in patients with CKD than in controls, and thiopental has altered pharmacokinetics with accumulation of free drug in the plasma; therefore, the rate of administration should be reduced in these patients. In regard to inhalation agents, methoxyflurane and enflurane can cause renal damage and should be avoided, whereas desflurane and isoflurane appear safer to use [87]. CKD is a risk factor for postoperative cardiac and renal complications, and consequently is associated with increased morbidity and mortality [87]; alterations of the pharmacokinetics and pharmacodynamics of anesthetics may cause more rapid peaks of plasma concentrations and decreased clearance, with a higher risk of side effects; thus, the dosages should be reduced, and prolonged infusions avoided [84].

Amide-type local anesthetics, like bupivacaine and ropivacaine, are mostly metabolized by the liver and are generally safe to use in patients with kidney failure; bupivacaine can also be used for nerve blocks [88], as these procedures are considered generally safe in CKD patients, though specific evidence is lacking [89]. In contrast, alterations of plasma binding proteins and metabolizing enzymes may reduce the effectiveness of lidocaine in patients with severe CKD [84]. In addition, pharmacokinetic alterations, combined with common metabolic CKD derangements like acidosis, hypoxia, and hypercarbia, can cause systemic toxicity even with local anesthetics, including seizures and cardiorespiratory alterations, and the patients should be closely monitored [84].

Another drug typically used for perioperative pain and anesthesia, clonidine, could also be effective for the treatment of chronic neuropathic pain associated with CKD, as seen in other conditions; intrathecal and epidural administration, in short-term infusions or with implantable pumps, provides effective long-term pain relief for patients with cancer, spinal cord injuries, or low back pain [90]. The effect is particularly evident if clonidine is associated with opioids or local anesthetics, as it appears to act as a co-analgesic rather than a pure analgesic. Furthermore, in dermal patches or gel, it significantly reduces diabetic neuropathic pain [90]. Clonidine is frequently prescribed to patients undergoing HD as an antihypertensive agent, although its safety in this population has not been studied extensively; reduced dosages are suggested by the manufacturer [91]. Further research is needed to assess the safety and efficacy of this drug for the treatment of chronic pain in patients with CKD.

**Figure 1:**
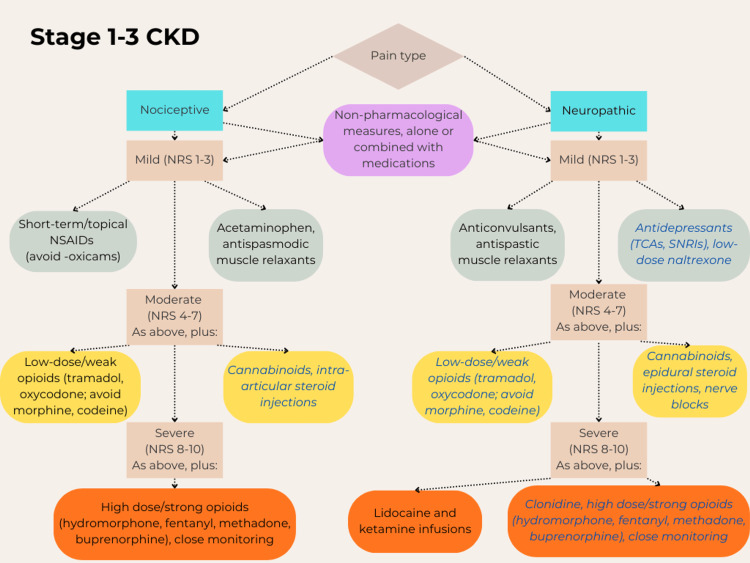
Steps for the treatment of chronic pain, from mild to severe, in patients with stage 1-3 of chronic kidney disease (CKD). Most of these drugs should be administered at reduced doses, except acetaminophen, the NSAIDs ibuprofen, indomethacin, and aspirin, the anticonvulsant carbamazepine, low-dose naltrexone, and ketamine [5,6,9,11,12,14-25,28-30,32-34,37,40,42-44,46-49,52-65,68-70,72-78,90,91]. Blue italics fonts: Drugs currently requiring further research for safety and/or efficacy. NRS: Numeric rating scale for pain intensity; NSAIDs: Non-steroidal anti-inflammatory drugs; TCAs: Tricyclic antidepressants; SNRIs: Serotonin-norepinephrine reuptake inhibitors. Author's own work.

**Figure 2:**
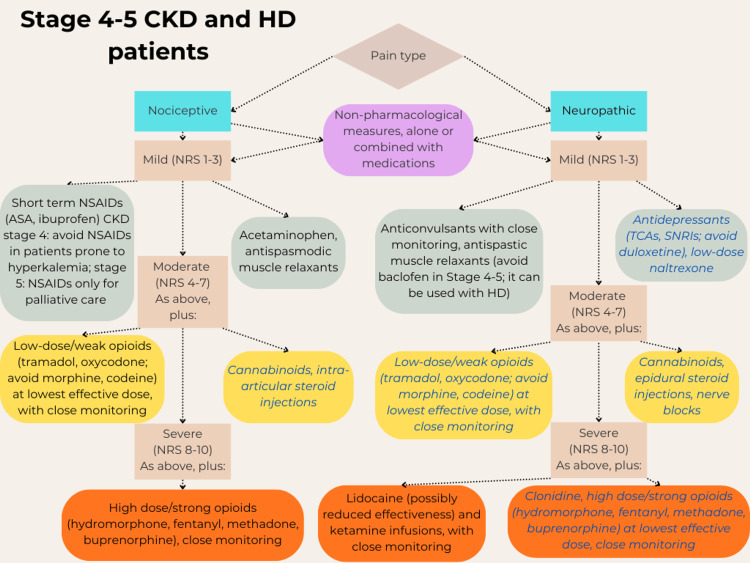
Steps for the treatment of chronic pain, from mild to severe, in patients with stage 4-5 of chronic kidney disease (CKD) and hemodialysis (HD) patients. Most of these drugs should be administered at reduced doses, except acetaminophen, the NSAIDs ibuprofen, indomethacin, and aspirin, the anticonvulsant carbamazepine, low-dose naltrexone, and ketamine [5,6,9,11,12,14-38,40-49,52-65,68-70,72-78,90,91]. Blue italics fonts: drugs currently requiring further research for safety and/or efficacy. NRS: Numeric rating scale for pain intensity; NSAIDs: Non-steroidal anti-inflammatory drugs; ASA: Acetylsalicylic acid (aspirin); TCAs: Tricyclic antidepressants; SNRIs: Serotonin-norepinephrine reuptake inhibitors. Author's own work.

**Figure 3:**
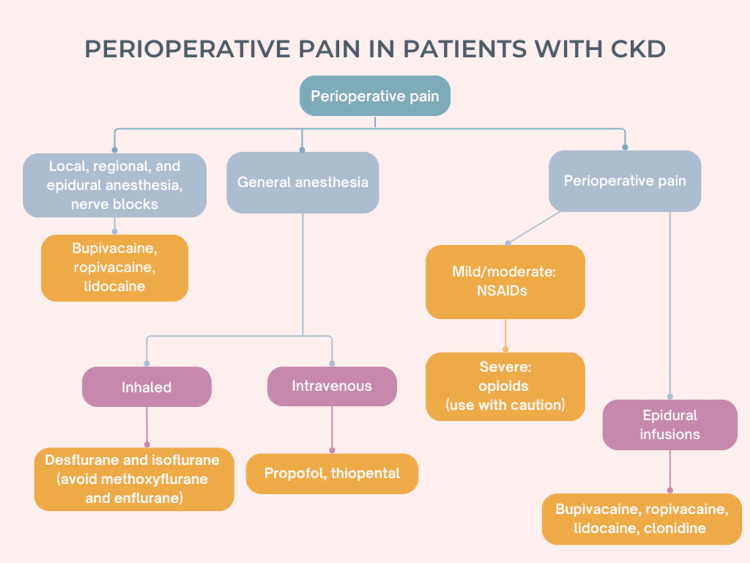
Options for anesthesia and perioperative pain in patients with chronic kidney disease (CKD). These drugs should be generally administered at reduced doses and with close monitoring of the patients. The local anesthetics lidocaine, bupivacaine, and ropivacaine are generally safer; however, monitoring is still necessary in case of metabolic derangements like acidosis, hypoxia, and hypercarbia [84-91]. NSAIDs: non-steroidal anti-inflammatory drugs. Author's own work.

**Figure 4:**
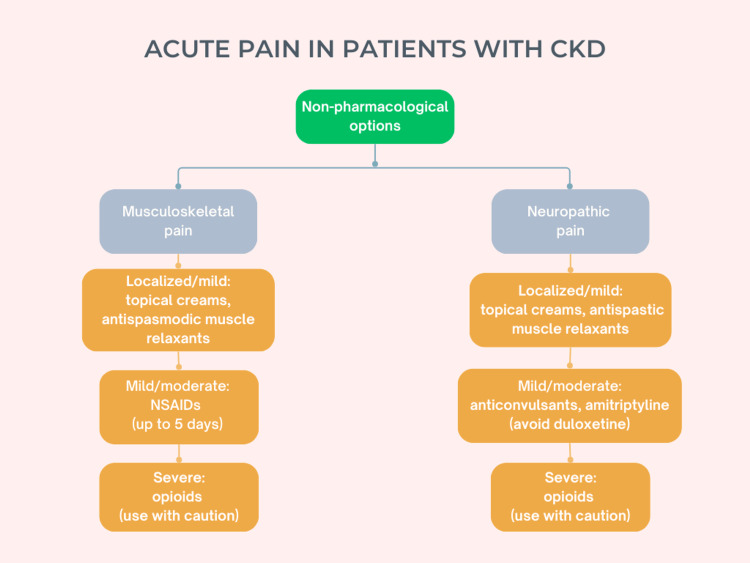
Steps for the treatment of acute pain, from mild to severe, in patients with chronic kidney disease (CKD). For acute pain, a step-wise approach can be followed from mild to severe pain, similar to the one for chronic pain [5,6,9,11,12,14-26,28-30,32-34,37,40,41]. NSAIDs: Non-steroidal anti-inflammatory drugs. Author's own work.

Finally, in case of acute pain, a ladder similar to the one already described for chronic pain can be followed [14,18]. Non-pharmacological options should be attempted first, like heat therapy or spinal manipulation for low back pain [18]. For common musculoskeletal injuries and minor postoperative pain, the most effective option is NSAIDs, at first topical and then oral if necessary, with or without diclofenac [18]. However, the latter should be used with caution, as this drug can increase the risk of major cardiovascular events in patients with CKD [26], in addition to the precautions already mentioned for NSAID use [19]. Opioids are not superior to NSAIDs for mild and moderate acute pain, and their use should be reserved for severe traumatic injuries, including crushes and burns, and pain after major surgeries, using immediate-release formulations at the lowest effective doses, only for the period of most severe pain, and on an as-needed basis rather than at scheduled intervals [18]. This regime helps reduce the risk of side effects and dependency, and the patient should be consulted and carefully informed of the risks and benefits of opioids before their use [18]. For acute neuropathic pain, the steps are similar to the corresponding chronic pain, with topical creams and antispastic muscle relaxants, anticonvulsants, and opioids for severe pain. Figures 1-4 summarize the various treatment options.

## Conclusions

Chronic pain is a common symptom in patients with CKD; it negatively affects QoL, morbidity, and mortality, and it is difficult to treat due to the altered pharmacokinetics and pharmacodynamics of several analgesics in these patients; thus, the dose should generally be lowered to reduce the risk of side effects. Pain management needs to be individualized, and the patient carefully monitored for side effects or insufficient pain relief, adjusting the dosage or changing medication as needed, or combining different approaches.

Non-pharmacological options can be attempted first, safe for all patients; additionally, in case of mild CKD and mild pain, short-term NSAIDs could be used, possibly adopting safer options like acetaminophen or topical formulations, and adjuvants like muscle relaxants, plus anticonvulsants and antidepressants for neuropathic pain, in case of prolonged requirements. If needed, mild or strong opioids (mostly effective for nociceptive pain) can be used in selected patients with moderate or severe pain, respectively. Generally, these medications should be administered starting at lower doses in patients with CKD than in subjects with preserved renal function to reduce the risk of dependency and side effects. In case of nociceptive pain inadequately controlled with acetaminophen or topical NSAIDs, the choice between oral NSAIDs or opioids should be based on a careful individualized patient analysis, including risk factors, age, CKD stage, comorbidities, and other medications needed by the patients, considering also the possible pharmacological interactions. A trial-and-error approach can help identify the most suitable drug(s) and the optimal dosage for effective pain control while minimizing the risks.

However, these options are often insufficient or used inappropriately, and patients frequently report inadequate pain management; therefore, further research and suitable alternatives are needed. For neuropathic pain, promising emerging alternatives include cannabinoids, low-dose naltrexone, and ketamine infusions, although larger and targeted studies are needed to assess their safety and efficacy in the CKD population. Other standard pain treatments that may be used with some adjustments in patients with CKD include anesthesia (general, spinal/epidural, regional, nerve block, and local) and cortisone injections, intra-articular or epidural, though their safety in these patients remains to be evaluated.
